# Diversity of *Citrullus colocynthis* (L.) Schrad Seeds Extracts: Detailed Chemical Profiling and Evaluation of Their Medicinal Properties

**DOI:** 10.3390/plants12030567

**Published:** 2023-01-26

**Authors:** Merajuddin Khan, Mujeeb Khan, Khaleel Al-hamoud, Syed Farooq Adil, Mohammed Rafi Shaik, Hamad Z. Alkhathlan

**Affiliations:** Department of Chemistry, College of Science, King Saud University, P.O. Box 2455, Riyadh 11451, Saudi Arabia

**Keywords:** plant extracts, *Citrullus colocynthis*, chemical profiling, biological activities, phytoconstituents

## Abstract

Seeds and fruits of *Citrullus colocynthis* have been reported to possess huge potential for the development of phytopharmaceuticals with a wide range of biological activities. Thus, in the current study, we are reporting the potential antimicrobial and anticancer properties of *C. colocynthis* seeds extracted with solvents of different polarities, including methanol (M.E.), hexane (H.E.), and chloroform (C.E.). Antimicrobial properties of *C. colocynthis* seeds extracts were evaluated on Gram-positive and Gram-negative bacteria, whereas, anticancer properties were tested on four different cell lines, including HepG2, DU145, Hela, and A549. All the extracts have demonstrated noteworthy antimicrobial activities with a minimum inhibitory concentration (MIC) ranging from 0.9–62.5 µg/mL against *Klebsiella planticola* and *Staphylococcus aureus*; meanwhile, they were found to be moderately active (MIC 62.5–250 µg/mL) against *Escherichia coli* and *Micrococcus luteus* strains. Hexane extracts have demonstrated the highest antimicrobial activity against *K. planticola* with an MIC value of 0.9 µg/mL, equivalent to that of the standard drug ciprofloxacin used as positive control in this study. For anticancer activity, all the extracts of *C. colocynthis* seeds were found to be active against all the tested cell lines (IC_50_ 48.49–197.96 µg/mL) except for the chloroform extracts, which were found to be inactive against the HepG2 cell line. The hexane extract was found to possess the most prominent anticancer activity when compared to other extracts and has demonstrated the highest anticancer activity against the DU145 cell line with an IC_50_ value of 48.49 µg/mL. Furthermore, a detailed phytoconstituents analysis of all the extracts of *C. colocynthis* seeds were performed using GC–MS and GC–FID techniques. Altogether, 43 phytoconstituents were identified from the extracts of *C. colocynthis* seeds, among which 21, 12, and 16 components were identified from the H.E., C.E., and M.E. extracts, respectively. Monoterpenes (40.4%) and oxygenated monoterpenes (41.1%) were the most dominating chemical class of compounds from the hexane and chloroform extracts, respectively; whereas, in the methanolic extract, oxygenated aliphatic hydrocarbons (77.2%) were found to be the most dominating chemical class of compounds. To the best of our knowledge, all the phytoconstituents identified in this study are being reported for the first time from the *C. colocynthis*.

## 1. Introduction

Folk medicine has long been dependent on plants, which are considered as a crucial source of bioceuticals for the treatment and prevention of innumerable diseases for generations [1,2]. Albeit immense progress in modern medicine, a huge chunk of population across the globe, and particularly, people of a low- income category, are still dependent on natural product-based traditional methods of treatment for curing a variety of ailments [3,4]. The heavy use of these treatment methods is mainly derived by ancient knowledge, local credence, effectiveness, and low-cost [5]. Indeed, the recent emergence of pandemics has greatly renewed interests in the application of natural products, including plant-based materials and their compounds as nutraceuticals [6,7,8]. Traditionally, plant materials are dried, crushed, or extracted to generate products that are often referred as botanical medicines, herbal medicines, or phytotherapeuticals [9,10]. In such a way, a variety of commercial drugs have been fabricated from plant sources, and indeed, in the discovery of novel pharmaceuticals, plant materials offer several benefits due to their abundance in nature and wide geographic distribution [11,12]. Contrary to the use of extracts, modern medicines mostly rely on single substances, to ensure consistent efficacy and quality [13]. Nevertheless, modern drug discovery methods are still largely dependent on the process of extraction from natural products, modification of currently applied phytotherapeuticals, design and synthesis of molecules mimicking phytoconstituents, etc. [14].

Notably, plants generate therapeutic secondary metabolites to protect themselves from harmful pathogenic microorganisms, insects, and other creatures, which are referred as phytochemicals [15,16]. Most of the phytochemicals often possess antimicrobial properties, due to which they are also capable of protecting humans and animals against several infectious diseases caused by microorganisms or toxins [17,18]. These phytochemicals include several classes of compounds, such as phytosterols, terpenoids, flavonoids, alkaloids, phenolics, carotenoids, organic acids and proteases inhibitors, etc., which possess natural therapeutic properties and offer the best template for future pharmaceutical development [19,20]. Phytochemicals are typically extracted using a variety of techniques, including Soxhlet extraction, maceration, supercritical fluid extraction, subcritical water, and ultrasound mediated extractions, etc. [21].

Among these, solvent extraction is the most popular technique, which is efficacious and easy to use, and thus, widely applied for the extraction of therapeutic secondary metabolites from plants and other natural resources [22,23]. In this technique, the contents and yield of secondary metabolites varies with the type of solvents used for the extraction; this is due to the difference in the polarities and other physicochemical properties of the solvents [24]. For example, polar solvents facilitate the isolation of phenolic constituents and their glycosidic derivatives and saponins etc.; meanwhile, non-polar solvents are typically used to extract fatty acids and steroids, etc. Moreover, temperature, extraction time, amount of solvent with respect to plant material, part of the plant used, as well as the preparation method of plant materials, also have a significant effect on the quality and quantity of the resulting secondary metabolites [25]. Often, these parameters also have a strong influence on the biological properties of phytoconstituents, which is well documented in several studies [26]. Indeed, scientists have usually adopted diverse extraction techniques, solvents, and other parameters to obtain a variety of different and effective bioactive compounds [27]. This is typically achieved by the comparison of the biological properties, including the antimicrobial and anticancer potential of the extract of same part of the plant extracted in different solvents [28].

For example, Chiavaroli et al., have extracted and screened the leaf and bark extract of *Rhizophora racemosa* G. Mey. using different solvents and extraction methods [29]. Among these extracts, the methanolic leave and bark extracts, which were obtained by both the homogenizer-assisted extraction and maceration extraction method, have demonstrated an abundance of phenolics, flavonoids, and other phenolic acids, due to which they exhibited effective radical scavenging, total antioxidant and reducing potential. Similarly, in our previous study, we have investigated the effect of extraction solvents on the biological potential of therapeutic secondary metabolites [30]. To do that, the plant material of *Artemisia judaica* was extracted using three different solvents, including hexane, chloroform, and methanol [30]. In continuation of our previous research, we have selected *Citrullus colocynthis*, which is an important medicinal plant and has been used for centuries in traditional medicine for the treatment of various ailments [31].

*C. colocynthis* is an herbaceous desert plant consisting of perennial roots and vine-like stems [32]. It belongs to the family Cucurbitaceae, and it is native to the arid sandy areas of West Asia, Arabia, tropical Africa, and the Mediterranean [33]. This plant contains a battery of biologically active substances, including glycosides, flavonoids, alkaloids, fatty acids, and essential oils, etc. [34]. Different parts of *C. colocynthis* have long been used for treating various diseases, such as its fruit pulp (dried), which is effective in treating indigestion and gastroenteritis, while its fruit is known to possesses antioxidant, antimicrobial, and anti-inflammatory properties [35,36]. Moreover, the other pharmacological potential of the *C. colocynthis* include anti-diabetic, anthelmintic, analgesic, anti-allergic, and anti-cancerous properties, etc. [37]. Therefore, to investigate the effect of solvents on the biological potential of *C. colocynthis*, in this study, the plant material was extracted using different solvents, such as, methanol (M.E.), hexane (H.E.), and chloroform (C.E.). The phytoconstituents of each extract was determined separately using gas chromatographic methods. In addition, the antimicrobial and anticancer properties of each extract were assessed individually against several microorganisms and cell lines, respectively.

## 2. Results and Discussions

### 2.1. Chemo-Profiling of Different Extracts

**Figure 1:**
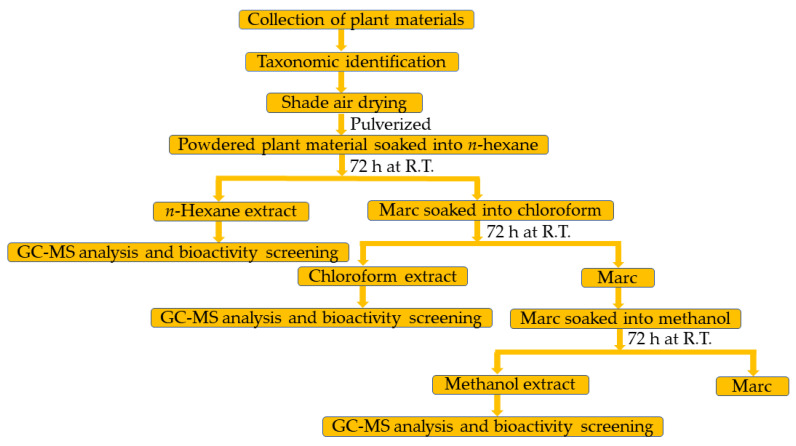
Flowchart for the preparation of *C. colocynthis* seeds extracts and their bioactivity screening.

Bioactive secondary metabolites are important for the physiology of both plants and humans, as they protect them by acting as anti-oxidants against oxidative stress [38]. In this regards, numerous studies have been reported in the literature describing the important biological properties of secondary metabolites including anti-microbial and anti-cancer properties [39,40]. Thus, the chemical characterization analysis of phytoconstituents of different extracts of the seeds of *C. colocynthis*, which are extracted with solvents of varied polarities, including methanol (M.E.), hexane (H.E.), and chloroform (C.E.), is undertaken. Moreover, the biological potentials including the antibacterial and anticancer properties of each individual extract were also evaluated. The solvent extractions of the seeds of *C. colocynthis* from Saudi Arabia was carried out at room temperature using conventional percolation technique, as described in earlier reports [30], and is shown in Figure 1.

The extraction was carried out individually by using an initial 500 g of seeds of *C. colocynthis* in each solvent, which yielded 11.2 g, 50.0 g, and 70.0 g of seed extract in M.E., H.E., and C.E., respectively. Notably, the extraction processes have yielded an almost similar color (dark brown) of extracts in all the solvents; however, their amount was slightly different due to the nature and quantity of the secondary metabolites extracted. For instance, C.E. extraction has resulted in higher yield, due to the high solubility of the long range of phytoconstituents including medium-polar and polar compounds in the chloroform solution. The chemo-profiling of all the extracts was carried out by GC–MS and GC–FID techniques, which has resulted in the recognition of a total of 21, 12, and 16 components from the H.E., C.E., and M.E. extracts, respectively. All the recognized phytochemicals generated from different extracts and their respective proportions are provided in the Table 1 based on the elution order of the compound from the column (HP-5MS).

According to the results in the Table 1, the H.E. extract was dominated by monoterpene hydrocarbons; whereas, the C.E. and M.E. extracts contained oxygenated monoterpenes and oxygenated aliphatic hydrocarbons as major chemical class of compounds, respectively. Particularly, the M.E. extract consisted of 77.2% of oxygenated aliphatic hydrocarbons; meanwhile, H.E. and C.E. extracts contained an almost similar amount of monoterpene hydrocarbons, i.e., 40.4% and oxygenated monoterpene hydrocarbons, i.e., 41.1%, respectively. Apart from the major chemical classes of phytoconstituents, each individual extract contains different chemical categories of compounds as subsequent chemical classes. For example, besides monoterpene hydrocarbons, the H.E. extract consisted of oxygenated aliphatic hydrocarbons and aromatics in an almost similar percentage, i.e., 19.3 and 21.5%, respectively. In the case of the C.E. extract, the oxygenated aliphatic hydrocarbons were present at a distant second position, which was recorded at 27.3%. In addition to these, the C.E. extract also contains diterpenoids, oxygenated sesquiterpenes, aliphatic hydrocarbons, and aromatics; however, chemical classes of these compounds were present in minor amount (<10% each). On the other hand, the M.E. extract does not contain other chemical classes of compounds in large quantities; after their major class of compounds, the other classes of phytoconstituents in M.E. extract are present in minor quantity just below 20% of total constituents. The categories of chemical compounds include oxygenated monoterpenes (3%), aliphatic hydrocarbons (4%), diterpenoids (9.6%), and others (<1%).

**Figure 2:**
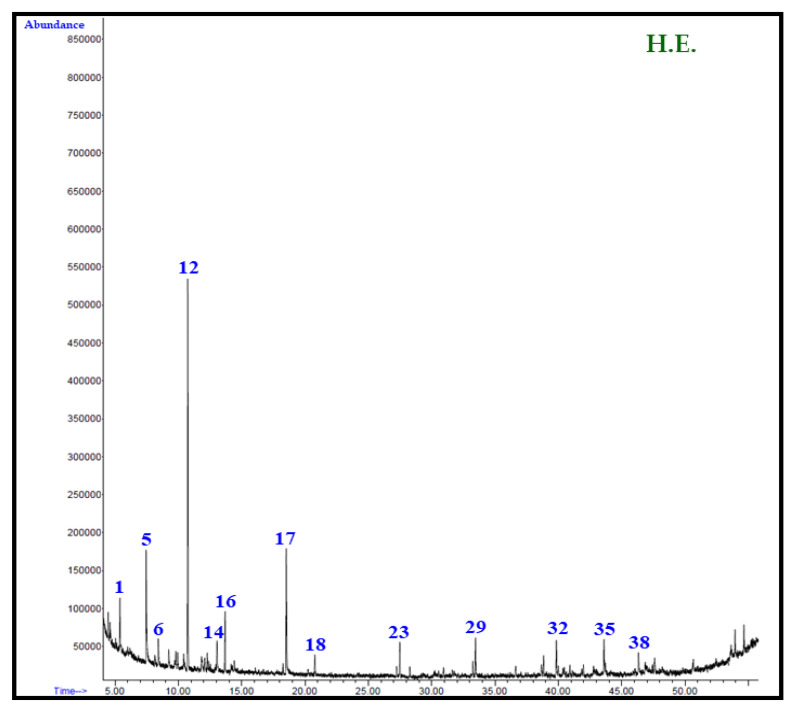

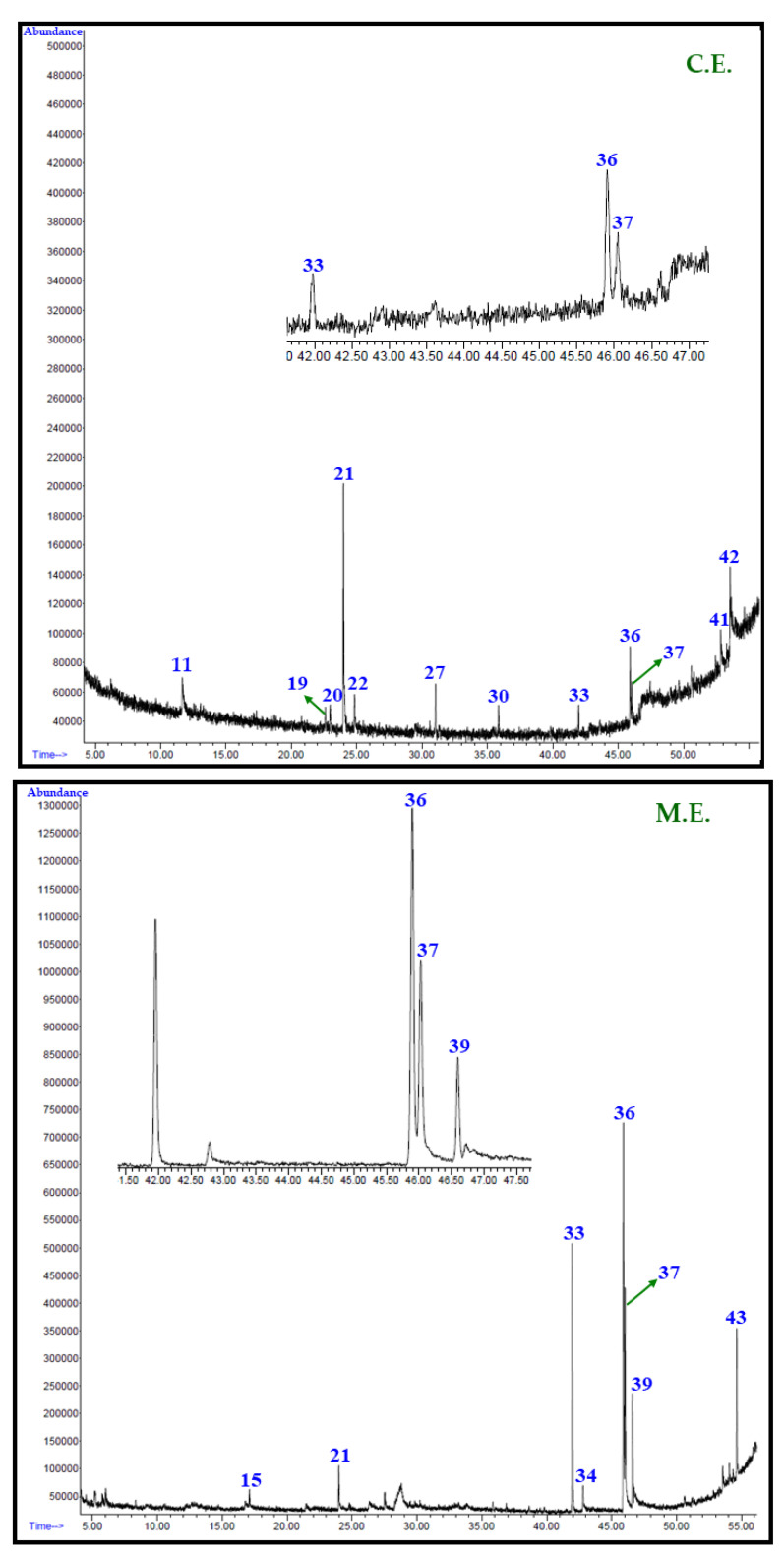
Total ion chromatogram (TIC) of *n*-hexane (H.E.), chloroform (C.E.), and methanol (M.E.) extracts of *C. colocynthis* seeds.

A comprehensive analysis of the phytoconstituents of all of the extract of seeds of *C. colocynthis* has revealed a total presence of 43 phytoconstituents; out of these components, 21, 12, and 16 components were identified from the H.E., C.E., and M.E. extracts, respectively. Among these, the H.E. extract clearly stands out with maximum number of phytoconstituents. The total ion chromatogram of each extracts of the *C. colocynthis* seed extracts are given in Figure 2. The H.E. extract was mostly dominated by α-pinene (30.6) followed by o-methylacetophenone (10.8%), isopropyl butanoate (10.4%), and *δ*3-carene (5.1%), while the remaining compounds, such as *p*-xylene, pseudocumene, tetradecane, hexadecane, methyl hexadecanoate, ethyl hexadecanoate, and methyl oleate are all present in a minor quantity of less than 5%. In the case of the C.E. extract, the major compound was identified as thymol (37.2%), which is followed by 8,11-octadecadienoic acid methyl ester (13.0%), *trans*-ferruginyl acetate (8.1%), and *β*-ionol (4.8%). The minor components of the same extract include filifolide-A (3.9%), ethyl phenyl acetate (3.1%), 2*E*-decenal (3.4%), 8-cedren-13-ol (2.8%), and tetracosane (2.1%). Whereas, the M.E. extract is mainly dominated by the 8,11-octadecadienoic acid methyl ester (28.6%) followed by the (*Z*)-9-octadecenoic acid methyl ester (20.4%), methyl hexadecanoate (18.3%), 6-ketoferruginol (9.6%), *n*-octadecanoic acid, methyl ester (7.4%), thymol (3.0%), and others, are present in an insignificant amount.

**Figure 3:**
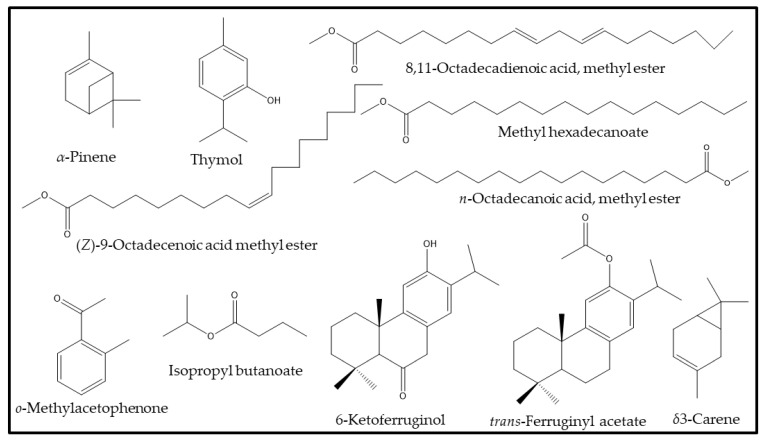
Chemical structure of most dominating identified compounds from *C. colocynthis* seeds extracts.

It is noteworthy that all the major compounds found in all three different extracts of *C. colocynthis* seeds, such as α-pinene, thymol, 8,11-octadecadienoic acid methyl ester and others (Figure 3), have been found to be distinct to the plant species collected from Riyadh region in KSA. These phytoconstituents have not been found in *C. colocynthis* collected from other regions of the world, as shown in Table 2. For example, the plant collected from the city of Tangier in Morocco has demonstrated the presence of nonadienal (15.4%), linalool propanoate (14.3%), and 2,4-decadienal (7.8%) as major constituents [41]. Whereas, the Indian species of the *C. colocynthis* collected from different cities have shown the presence of 2-methyl,4-heptanone (48.0), 3-methyl,2-heptanone (12.9), *n*-hexadecanoic acid (12.4), and morphine (9.1) as dominant compounds [42,43]. Particularly, the three major compounds found in each separate extract, such as α-pinene, thymol, and 8,11-octadecadienoic acid methyl ester in H.E., C.E., and M.E. extracts, respectively, have been known to possess excellent biological properties. These compounds are distinct to the plant species investigated in this study, and thus the seeds extract of the *C. colocynthis* collected from Riyadh are expected to demonstrate decent biological properties when compared to the same species gathered from other regions of the world.

Typically, α-pinene is an important secondary metabolite, which is mainly found in essential oils from different plants, such as the *Piper nigrum* or *Juniperus* species [47]. α-pinene is a monoterpene, which consists of hydrophobic and volatile properties with fresh pine scent and woody flavour [48]. This compound has been known to possess excellent antimicrobial properties against various Gram-positive and Gram-negative bacterial strains, including the methicillin-resistant *Staphylococcus aureus* [49]. Additionally, α-pinene has been reported to demonstrate anticancer properties against the human ovarian cancer PA-1 cell line [50]. On the other hand, thymol, which is a monoterpene phenol mainly found in essential oils of the plants from Lamiaceae family (*Thymus*, *Ocimum*, *Origanum*, and *Monarda* genera) is also surprisingly found in the C.E. extract of the *C. colocynthis* seeds [51]. Mainly, the thymol-based plant species are used as flavouring and preservative agents and has also been recognized as “safe” (GRAS) or as approved food additives [52]. Thymol is known to possesses excellent anti-inflammatory, anti-microbial, anti-oxidant, and antifungal properties, besides being beneficial for the cardiovascular system [53]. The solvent-based variation in major constituents is not new. Indeed, plants demonstrate a huge difference in their phytochemical constituents, which are typically based on a variety of different factors, such as geographic location, genetic variations, ecological and environmental factors, etc. [39,54]. Similarly, different experimental conditions, such as the solvent, temperature, and pH of extraction processes also have serious effects on the quality and quantity of the phytomolecules. The difference in the major constituents may possibly have different synergistic interactions, which ultimately determine the biological activities of plant-based materials [55].

For example, in a recent study, the monoterpenes’ thymol demonstrated direct antibacterial activity against the *S. aureus* IS-58 strain [56]. Additionally, thymol has also been recognized as anti-tumor agent, which is demonstrated in a recent study during the evaluation of the cell viability and apoptosis in U-87 cells treated with thymol at different concentrations. The half-maximal inhibitory concentration (IC_50_) of thymol in the U-87 cells was 230 μM, while on a normal cell line it did not exhibit any cytotoxic effect at the same concentration [57]. Besides the two biologically active phytoconstituents, α-pinene and thymol, another major constituent, 8,11-octadecadienoic acid, as the methyl ester found in M.E. extract, has also demonstrated excellent biological properties in previously reported studies [58]. For example, the ethyl acetate extract of the seeds of *Acacia nilotica* Linn, which contained 11-Octadecenoic acid, methyl ester as major compound, has demonstrated excellent activities against the several tested microbes with zones of inhibition diameters ranging from 27–32 mm against *Salmonela* typhi, *E. coli*, *Streptococcus feacalis*, *S. aureus,* and so on [59].

### 2.2. Antibacterial Properties

In order to test the antimicrobial efficacy of seeds’ extracts of *C. colocynthis*, all the different extracts, including H.E., C.E., and M.E. extracts, were employed against both Gram-positive bacterial strains, such as *S. aureus* and *M. luteus,* and Gram-negative bacterial strains, such as *K. planticola* and *E. coli*, respectively. Whereas, ciprofloxacin, which is commonly prescribe as antibiotic, has been used as a control during this study. The extracts have delivered mixed results, i.e., the extracts were active against both Gram-positive and Gram-negative bacterial strains; however, neither of them demonstrated good activities against *E. coli,* which is Gram-negative bacteria. For instance, both H.E. and C.E. extracts were active against *S. aureus* and *K. planticola*, which are Gram-positive and Gram-negative bacteria, respectively. Whereas, the M.E. extract demonstrated antibacterial activity only against a single bacterial strain, i.e., *K. planticola*. Among these extracts, the H.E. extract has demonstrated superb antibacterial activity against *K. planticola*, which is almost comparable to the commercially available antibiotic. Meanwhile, the other extracts demonstrated very mild activities towards the tested strains. 

The results further revealed that the C.E. extract exhibited mild activity against *S. aureus* with 7.8 μg/mL, but demonstrated excellent potential towards *K. planticola*, with 1.9 μg/mL. Meanwhile, the H.E. extract exhibited decent activity against *S. aureus* with 3.9 μg/mL; whereas, it demonstrated the best of all antibacterial activities against the *K. planticola*, with 0.9 μg/mL, which was equal to the activity of the commercially available antibiotic, i.e., ciprofloxacin (cf. Table 3). On the other hand, the M.E. was the least active extract among the tested materials, and demonstrated decent activity towards a single strain, which is 7.8 μg/mL towards the *K. planticola*. Notably, all the extracts studied have demonstrated very mild activity against the *M. luteus* and *E. coli*, except the slightly decent activity of the M.E. extract against *M. luteus,* with 62.2 μg/mL.

Among all the extracts, the best antimicrobial activity was demonstrated by he H.E. extract against *K. planticola,* with 0.9 μg/mL, which is same as the commercially available antibiotic (ciprofloxacin). Similarly, the decent antibacterial activity of the H.E. extract was also observed against *S. aureus,* with 0.9 μg/mL. However, the same extract demonstrated very mild activity against the other two species of bacteria, namely *M. leuteus* and *E. coli*. These results are not surprising, as the H.E. extract contains high amount of α-pinene, which is already known to exhibit strong antibacterial activities against several bacterial strains, including *K. planticola* and *S. aureus* [60]. For example, the essential oil of *Baccharis reticulata,* which contains a high amount of α-pinene, demonstrated excellent antibacterial activities against *S. aureus* with a MIC value of 256 μg/mL; whereas, the same extract did not have a significant effect on *E. coli* and other bacteria [61]. α-pinene in a pure form demonstrates excellent antibacterial activities against a large number of bacterial strains; however, when present in the extract or essential oils together with other phytoconstituents, it selectively targets the bacterial species. This can be attributed to the presence of antagonist phytoconstituents, which may stop the action or effect of α-pinene. As in the H.E. extract, besides α-pinene, isopropyl butanoate, *o*-methylacetophenone, and many other phytochemicals are present, which may function as antagonist phytomolecules. Similar results were also obtained the in case of the C.E. extract, which consisted of thymol as the major constituent, and is already known to have demonstrated excellent antibacterial properties towards several strains [62]. For example, thymol-rich essential oils of *Oliveria decumbens* (Apiaceae) collected from different Iranian populations demonstrated high antibacterial properties against a variety of Gram-positive and Gram-negative bacteria. The essential oils obtained from *Oliveria decumbens* (Apiaceae) collected from the Behbahan region of Iran exhibited a MIC value of 1.0 μg/mL against *S. aureus* (Gram-positive), while the thymol-rich C.E. in this study demonstrated a near similar antibacterial property with a MIC value of 7.8 μg/mL [63]. The gas chromatographic-mass spectrometry analysis put in evidence four main volatile constituents, such as thymol (20.3–36.4%), However, very little has been published with regards to the biological properties of 8,11-Octadecadienoic acid, methyl ester, which is a major constituent of M.E. extract. This is also reflected in our study, where the M.E. extract has demonstrated the least antibacterial activities when compared to the other extracts.

### 2.3. Anticancer Properties

Besides antibacterial properties, the seed extracts of *C. colocynthis* were also evaluated for their potential anticancer properties, which is explored against a variety of cell lines, including HepG2 (hepatic cancer cells), DU145 (prostate cancer cells), Hela (cervical cancer cells), and A549 (human lung cancer cells). During this study, doxorubicin, a prescription anticancer drug, was employed as a control, which is a commercially available anticancer drug (cf. Table 4). All the studied extracts have demonstrated diverse anticancer activities against different cell lines, which are provided in Table 4. When compared to the controlled drug used in this study, which has shown IC_50_ (µg/mL) of less than one (<1) against all the studied cell lines, neither of the extracts has demonstrated the activity, which is close to the value of doxorubicin. The M.E. extract has exhibited IC_50_ values of 126.6, 91.9, 99.9, 70.1 µg/mL against HepG2, DU145, Hela, and A549, respectively. Whereas, the H.E. and C.E. extracts have demonstrated the IC_50_ values of 177.0 and no activity, and 48.4 and 53.3, 197.2 and 83.8, 82.9 and 154.0 µg/mL against the same cell lines, respectively. However, the IC_50_ values are insignificant when compared to the controlled drug, and, according to a reported study on the extensive screening of several extracts from a variety of plants, a plant extract is usually considered to possess in vitro cytotoxic activity when the IC_50_ (concentration that causes a 50% cell kill) value is less than 20 µg/mL for the extract [64].

Taking this into account, only the H.E. and C.E. extract has demonstrated very mild 48.4 and 53.3 µg/mL, respectively, against a single cell line, namely, DU145. Nevertheless, the major constituents of these extracts, including α-Pinene and thymol, have been reported to demonstrate considerable anticancer properties against a battery of cell lines. For example, pine needle oil from the crude extract of pine needles, which consists of large amount of α-Pinene, has exhibited considerable inhibitory effect on hepatoma carcinoma BEL-7402 cells, with an inhibitory rate of 79.3% in vitro and 69.1% in vivo [65]. Similarly, the crude extract of *Trachyspermum ammi* consisting of thymol in large amount has also shown potential cytotoxic activity in the breast cancer cell line MCF-7. The MTT assay demonstrated that the IC_50_ values of thymol on MCF-7 cells for 48 h and 72 h were 54 and 62 μg/mL, respectively [66]. These values are close to the IC_50_ values of the C.E. extract of *C. colocynthis* seeds (53.2 µg/mL), which has thymol as major constituents.

Despite the mild anticancer properties of all the studied extracts against different cancer cell lines, the data still demonstrate a clear trend for the selection of extracts for the activity guided isolation of phytomolecules, which is essential in the quest of finding biologically active phytoconstituents [67]. In this regard, no prior reports on the comprehensive analysis of the anticancer properties of the seed extracts of *C. colocynthis* in different solvents with varying polarities have been reported in the literature. However, few reports have appeared on the anticancer potential of the essential oils of the seed of *C. colocynthis*, which have demonstrated reasonable anticancer properties towards colorectal cancer cell lines, with IC_50_ values varying between 4 and 7 mg/mL [68]. Meanwhile, the other studies have reported that the seed and pulp extracts (extracted using a single solvent) of the fruit of *C. colocynthis* were effective against various cancer cell lines [69]. However, in this study, we have employed three different solvents with varying polarities to isolate the extracts of the *C. colocynthis* seeds, which have delivered notable results with different major constituents in a different solvent extract. Similar to the antibacterial results, the M.E. extract with 8,11-Octadecadienoic acid, methyl ester as major constituent has not demonstrated decent anticancer activities with the IC_50_ values of more 75 μg/mL against almost all the cell lines studied.

## 3. Materials and Methods

### 3.1. Plant Material

Entire aerial parts of the *C. colocynthis* grown in the region of Taif, a city in the Mecca Province of southwest Saudi Arabia, were procured in May 2020. Identifications of *C. colocynthis* were authenticated by Dr. Rajakrishnan Rajagopal from the herbarium division of King Saud University. A specimen sample (24,531) of *C. colocynthis* is retained in the herbarium division of the King Saud University.

### 3.2. Chemicals

All the chemicals including methanol, chloroform, and *n*-hexane were of analytical grade and purchased from Sigma–Aldrich, Hamburg, Germany. Pure volatile constituents or enriched fractions of volatile constituents, such as thymol, *δ*3-carene, *α*-pinene (Alfa Aesar, Lancashire, UK), *n*-hexadecanoic acid, caryophyllene oxide, (*Z*)-9-Octadecenoic acid methyl ester, and 8,11-Octadecadienoic acid, methyl ester (enriched fractions), were available and used for co-injection/comparative analysis.

### 3.3. Preparation of C. colocynthis Seeds Extracts

Procured aerial parts of *C. colocynthis* were air-dried at room temperature. The fruits, leaves, and stem of the plant were separated and subjected to drying separately until a constant weight was achieved. From the fruits of *C. colocynthis*, the seeds were carefully removed and then ground using a grinder. The resultant *C. colocynthis* seeds (500 g) were first percolated with *n*-hexane (550 mL) three times at room temperature. After *n*-hexane extraction, the marc was again subjected to extraction three times with CHCl_3_ (550 mL). Finally, the same extraction process was repeated using the residual marc with methanol (550 mL) for three times at room temperature. Notably, each time, the extraction process was carried out for 3 days for all the solvents employed. The resultant *n*-hexane, chloroform, and methanol extracts of *C. colocynthis* seeds were separately dried under a vacuum at 40 °C until the solvents were completely removed using a Buchi rotary evaporating system (Rotavapor R-215, Buchi, Flawil, Switzerland) equipped with a vacuum controller (V-850) and vacuum pump (V-700). These separately dried *n*-hexane, CHCl_3_, and methanol extracts were used for the screening of anticancer and antimicrobial activities, as well as for the GC analysis.

### 3.4. GC and GC–MS Analysis of C. colocynthis Seeds Extracts

In order to identify the chemical constituents of the extracts of *C. colocynthis* seeds, the dried extracts of *n*-hexane and CHCl_3_ extracts were dissolved in diethyl ether, whereas the methanol extract was dissolved in methanol and subjected to GC–FID and GC–MS analyses. The GC–MS system was equipped with stationary phase columns (HP-5MS) employing the same method, as described previously [70]. Detailed methodology is given in Supplementary Materials (Scheme S1). The identified constituents from *n*-hexane, CHCl_3_, and methanol extracts of *C. colocynthis* seeds and their relative percentages are provided in Table 1, and the constituents are listed according to their elution order on the HP-5MS column.

### 3.5. Calculation of Linear Retention Indices (LRIs)

LRI values of chemical constituents of *C. colocynthis* seeds extracts were determined following a previously reported method [70], and they are listed in Table 1. The detailed methodology is provided in Supplementary Materials (Scheme S2).

### 3.6. Identification of Volatile Chemical Components

Identification of the chemical constituents of *C. colocynthis* seeds extracts were carried out through an analysis on a HP-5MS column, as described previously [70]. Detailed methodology for the identification of chemical constituents is provided in the Supplementary Materials (Scheme S3) [71,72,73]. GC–MS chromatograms for the identified constituents of *n*-hexane, chloroform, and methanol extracts of *C. colocynthis* seeds on the HP-5MS column are given in Figure 2.

### 3.7. Evaluation of Antimicrobial and Anticancer Activity

#### 3.7.1. Antimicrobial Activity

Antimicrobial activity of the *C. colocynthis* seeds extracts was examined using the well diffusion method [74] towards a panel of four pathogenic bacterial strains, including *Staphylococcus aureus* MTCC 96, *Micrococcus luteus* MTCC 2470, *Escherichia coli* MTCC 739, and *Klebsiella planticola* MTCC 530. The four pathogenic reference strains were spread on the surface of the Mueller–Hinton agar Petri plates with 0.1 mL of previously prepared microbial suspensions individually containing 1.0 × 10^7^ CFU/mL (equal to 0.5 McFarland standard). Using a cork borer, the wells of the 6.0 mm diameter were prepared in the media plates, and the prepared test extracts at a dosage range of 250–0.48 µg/well were added in each well under sterile conditions in a laminar air flow chamber. The standard antibiotic solution of Ciprofloxacin at a dose range of 250–0.48 µg/well and the well containing dimethyl sulfoxide (DMSO) served as positive and negative controls, respectively. The plates were incubated for 24 h at 37 °C, and the well containing the least concentration showing the inhibition zone was considered as the minimum inhibitory concentration (MIC). All experiments were carried out in duplicates and mean values are represented.

#### 3.7.2. Anticancer Activity

Cytotoxicity of *C. colocynthis* seeds extracts was assessed against the human lung adenocarcinoma cell line (A549), human hepatocarcinoma cell line (HepG2), human cervical cancer cell line (HeLa), and human prostate cancer cell line (DU145) using the MTT assay [75]. Briefly, 1 × 10^4^ exponentially growing cells were seeded into each 96-well plate (counted by Trypan blue exclusion dye method) and allowed to grow until 60–70% confluence; then, different concentrations of test extracts were added to the culture medium along with negative (DMSO) and positive controls (Doxorubicin). The plates were incubated for 48 h in a CO_2_ incubator at 37 °C with a 90% humidified atmosphere and 5% CO_2_. Then, the media of the wells were replaced with 90 µL of fresh serum-free media and 10 µL of MTT (5 mg/mL of PBS), and the plates were incubated at 37 °C for 2 h. The media was discarded and allowed to dry for 30 min. Later, 100 µL of DMSO was added in each well to dissolve the purple formazan crystals, and the absorbance was recorded at 570 nm using Spectra Max plus 384 UV-Visible plate reader (Molecular Devices, Sunnyvale, CA, USA). Each test compound was examined at various concentrations in triplicate, and the results are expressed as a mean with standard deviation (mean ± SD), (*n =* 3). One-way ANOVA and Dunnett’s post-comparison test were used to analyse the data for significant differences (test vs. control). The statistical significance for the experiment was set at *p* < 0.05.

## 4. Conclusions

In this study, the effect of the polarity of the extraction solvents on the phytochemical contents and biological potential of the extracts of the seeds of *C. colocynthis* were explored. To do this, three different solvents, including M.E., C.E., and H.E., were selected to isolate the phytoconstituents of the studied plant material. The contents of all the studied extracts were vastly different with respect to their major components, and the H.E. and C.E. extracts demonstrated *α*-pinene and thymol as their major constituents, respectively; whereas, the M.E. extract demonstrated the presence of 8,11-octadecadienoic acid, methyl ester in a large quantity. Out of all the extracts, the H.E. and C.E. extracts clearly stood out in terms of their major constituents and their biological potential. Particularly, the H.E. extract, consisting of *α*-pinene (30.6%), demonstrated a superior antimicrobial activity against most of the strains studied and indeed, in the case of *K. planticola,* it demonstrated excellent antibacterial activity, which was almost equal to the commercially available antibiotic. Therefore, the *C. colocynthis* seed extracts may offer a variety of phytopharmaceutical, food products, and other commercial entities in the form of biologically active pure phytomolecules such as *α*-pinene and thymol.

## Figures and Tables

**Table 1:** Identified chemical constituents from various extracts of *C. colocynthis* seeds of Saudi Arabia.

Peaks	Compounds *	M.F	CAS No.	R.T. (min)	LRI	H.E. %	C.E. %	M.E. %
1	*cis*-2-Pentenol	C_5_H_10_O	1576-95-0	5.187	772	-	-	0.7
2	Toluene	C_7_H_8_	108-88-3	5.394	778	3.6	-	-
3	Capronaldehyde	C_6_H_12_O	66-25-1	5.996	796	-	-	0.2
4	1-Octene	C_8_H_16_	111-66-0	6.034	797	-	-	0.4
**5**	**Isopropyl butanoate**	**C_7_H_14_O_2_**	**638-11-9**	**7.466**	**840**	**10.4**	**-**	**-**
6	*p*-Xylene	C_8_H_10_	106-42-3	8.422	869	2.8	-	-
7	*o*-Xylene	C_8_H_10_	95-47-6	9.251	894	1.6	-	-
8	Santolina triene	C_10_H_16_	2153-66-4	9.806	909	1.6	-	-
9	Isobutyl isobutyrate	C_8_H_16_O_2_	97-85-8	9.958	913	1.6	-	-
10	*α-*Thujene	C_10_H_16_	2867-05-2	10.43	926	1.5	-	-
11	Benzaldehyde	C_7_H_6_O	100-52-7	11.662	958	-	1.6	-
**12**	***α*-Pinene**	**C_10_H_16_**	**80-56-8**	**10.754**	**934**	**30.6**	**-**	**-**
13	Sabinene	C_10_H_16_	3387-41-5	12.29	974	1.6	-	-
14	Pseudocumene	C_9_H_12_	95-63-6	13.058	994	2.7	-	-
15	Undecane	C_11_H_24_	1120-21-4	17.098	1100	-	-	1.1
**16**	***δ*3-Carene**	**C_10_H_16_**	**13466-78-9**	**13.696**	**1011**	**5.1**	**-**	**-**
**17**	***o*-Methylacetophenone**	**C_9_H_10_O**	**577-16-2**	**18.527**	**1138**	**10.8**	**-**	**-**
18	Dodecane	C_12_H_26_	112-40-3	20.783	1200	1.4	-	-
19	Ethyl phenyl acetate	C_10_H_12_O_2_	101-97-3	22.604	1253	-	3.1	-
20	*2E*-Decenal	C_10_H_18_O	3913-81-3	22.971	1263	-	3.4	-
**21**	**Thymol**	C_**10**_H_**14**_**O**	**499-75-2**	**23.988**	**1293**	**-**	**37.2**	**3.0**
22	Filifolide-A	C_10_H_14_O_2_	-	24.83	1318	-	3.9	-
23	Tetradecane	C_14_H_30_	629-59-4	27.483	1400	2.8	-	1.5
24	Coumarin	C_9_H_6_O_2_	91-64-5	28.542	1434	-	-	0.3
25	2-Methyl butyl benzoate	C_12_H_16_O_2_	52513-03-8	28.696	1439	-	-	0.5
26	*α*-Guaiene	C_15_H_24_	3691-12-1	28.795	1443	-	-	0.2
27	*β*-Ionol	C_13_H_22_O	22029-76-1	31.038	1517	-	4.8	-
28	Caryophyllene oxide	C_15_H_24_O	1139-30-6	33.252	1593	1.4	-	-
29	Hexadecane	C_16_H_34_	544-76-3	33.459	1600	3.1	-	-
30	8-Cedren-13-ol	C_15_H_24_O	18319-35-2	35.846	1686	-	2.8	-
31	Octadecane	C_18_H_38_	593-45-3	38.831	1800	1.7	-	-
32	7-Hydroxycoumarin	C_9_H_6_O_3_	93-35-6	39.837	1840	3.9	-	-
**33**	**Methyl hexadecanoate**	C_**17**_H_**34**_O_**2**_	**112-39-0**	**41.982**	**1927**	**-**	**5.6**	**18.3**
34	*n*-Hexadecanoic acid	C_16_H_32_O_2_	57-10-3	42.789	1960	-	-	1.6
35	Ethyl hexadecanoate	C_18_H_36_O_2_	628-97-7	43.603	1994	3.3	-	-
**36**	**8,11-Octadecadienoic acid, methyl ester**	C_**19**_H_**34**_O_**2**_	**56599-58-7**	**45.91**	**2091**	**-**	**13.0**	**28.6**
**37**	(***Z*****)-9-Octadecenoic acid methyl ester**	C_**19**_H_**36**_O_**2**_	**112-62-9**	**46.029**	**2096**	**-**	**5.3**	**20.4**
38	Methyl oleate	C_19_H_36_O_2_	112-62-9	46.319	2108	2.1	-	-
**39**	***n*** **-Octadecanoic acid, methyl ester**	C_**19**_H_**38**_O_**2**_	**112-61-8**	**46.599**	**2120**	**-**	**-**	**7.4**
40	Ethyl linoleate	C_20_H_36_O_2_	544-35-4	47.605	2162	1.9	-	-
41	Tetracosane	C_24_H_50_	646-31-1	53.297	2400	-	2.1	1.4
**42**	***trans*** **-Ferruginyl acetate**	C_**22**_H_**32**_O_**2**_	**15340-79-1**	**53.562**	**2411**	**-**	**8.1**	**-**
**43**	**6-Ketoferruginol**			**54.625**	**2456**	**-**	**-**	**9.6**
Monoterpenes hydrocarbons						40.4	-	-
Oxygenated monoterpenes						-	41.1	3.0
Sesquiterpene hydrocarbons						-	-	0.2
Oxygenated sesquiterpenes						1.4	7.6	-
Aliphatic hydrocarbons						9	2.1	4.4
Oxygenated aliphatic hydrocarbons						19.3	27.3	77.2
Aromatics						21.5	4.7	0.5
Diterpenoids						-	8.1	9.6
Others						3.9	-	0.3
Total identified						**95.5**	**90.9**	**95.2**

* Components are recorded as per their order of elution from HP-5MS column; compounds higher than 5.0% are highlighted in boldface; LRI = linear retention index computed with reference to the *n*-alkanes mixture (C7–C30) on HP-5MS column; H.E. = hexane extract of *C. colocynthis* seeds; C.E. = chloroform extract of *C. colocynthis* seeds; M.E. = methanol extract of *C. colocynthis* seeds.

**Table 2:** Major components of *C. colocynthis* from different parts of the world.

No.	Country	City	Major Components (%)	Reference
1.	Morocco	Tangier	Nonadienal (15.4), linalool propanoate (14.3), 2,4-decadienal (7.8), pentadecane (7.2), hexanal (4.5), and butylated hydroxy anisol (4.3).	[44]
2.	India	Parangipettai	2-Methyl,4-heptanone (48.0), 3-Methyl,2-heptanone (12.9), trimethylsilylmethanol (9.1), pentane, 1-propoxy (6.5), and 2-pentanol, 5-methoxy-2-methyl- (5.3).	[45]
		Southern Haryana	*n*-Hexadecanoic acid (12.4), morphine (9.1), narceine (10.3), isoquinoline, 1-[(3,4-dimethoxyphenyl)methyl]-6,7-dimethoxy- (7.5), codein (6.6), and glycerol (5.5).	[46]
3.	Saudi Arabia	Riyadh	Thymol (3.0–37.2)*, α*-pinene (0–30.0), 8,11-octadecadienoic acid, methyl ester (13.0–28.6), *(Z)*-9-octadecenoic acid methyl ester (0–20.4), methyl hexadecanoate (5.6–18.3), *o*-methylacetophenone (0–10.8), isopropyl butanoate (0–10.4), 6-ketoferruginol (0–9.6), *trans*-ferruginyl acetate (0.0–8.1), *n*-octadecanoic acid, methyl ester (0–7.4), *trans*-sabinyl acetate (0–6.0).	Present study

**Table 3:** Antimicrobial activity of various extracts of *C. colocynthis* seeds against Gram-positive and Gram-negative bacteria.

Tested Extracts of *C. colocynthis* Seeds	Minimum Inhibitory Concentration (µg/mL)			
	Gram-Positive		Gram-Negative	
	*S. aureus* MTCC 96	*M. luteus* MTCC 2470	*K. planticola* MTCC 530	*E. coli * MTCC 739
M.E.	62.5	62.5	7.8	>250
H.E.	3.9	>250	0.9	>250
C.E.	7.8	>250	1.9	>250
Ciprofloxacin	0.9	0.9	0.9	0.9

**Table 4:** Anticancer activity of various extracts of *C. colocynthis* seeds against various cancer cell lines.

Tested Extracts of *C. colocynthis* Seeds	IC_50_ (µg/mL)			
	HepG2	DU145	Hela	A549
M.E.	126.65 ± 11.48	91.94 ± 7.88	99.96 ± 9.70	70.18 ± 1.17
H.E.	177.05 ± 4.84	48.49 ± 0.50	197.28 ± 9.45	82.99 ± 6.5
C.E.	NA	53.32 ± 1.59	83.87 ± 4.61	154.05 ± 14.25
Doxorubicin	0.72 ± 0.012 (µM)	0.36 ± 0.01 (µM)	0.8 ± 0.71 (µM)	0.55 ± 0.16 (µM)

Results are expressed as mean ± SD, NA = No activity.

## Data Availability

Data are contained within the article.

## Supplementary Materials

The following supporting information can be downloaded at: https://www.mdpi.com/article/10.3390/plants12030567/s1. Scheme S1. Gas Chromatography (GC) and Gas Chromatography−Mass Spectrometry (GC-MS) Analysis of *C. colocynthis* seeds Extracts; Scheme S2. Linear retention indices (LRIs); Scheme S3. Identification of volatile components.

## References

[1] Makhuvele R., Naidu K., Gbashi S., Thipe V.C., Adebo O.A., Njobeh P.B. (2020). The use of plant extracts and their phytochemicals for control of toxigenic fungi and mycotoxins. Heliyon.

[2] Junsongduang A., Kasemwan W., Lumjoomjung S., Sabprachai W., Tanming W., Balslev H. (2020). Ethnomedicinal knowledge of traditional healers in Roi Et, Thailand. Plants.

[3] Uttra A.M., Ahsan H., Hasan U.H., Chaudhary M.A. (2018). Traditional medicines of plant origin used for the treatment of inflammatory disorders in Pakistan: A review. J. Tradit. Chin. Med..

[4] Zhao Z., Li Y., Zhou L., Zhou X., Xie B., Zhang W., Sun J. (2021). Prevention and treatment of COVID-19 using Traditional Chinese Medicine: A review. Phytomedicine.

[5] Tesfaye S., Asres K., Lulekal E., Alebachew Y., Tewelde E., Kumarihamy M., Muhammad I. (2020). Ethiopian medicinal plants traditionally used for the treatment of cancer, part 2: A review on cytotoxic, antiproliferative, and antitumor phytochemicals, and future perspective. Molecules.

[6] Khan M., Adil S.F., Alkhathlan H.Z., Tahir M.N., Saif S., Khan M., Khan S.T. (2020). COVID-19: A global challenge with old history, epidemiology and progress so far. Molecules.

[7] Pastor N., Collado M.C., Manzoni P. (2021). Phytonutrient and nutraceutical action against COVID-19: Current review of characteristics and benefits. Nutrients.

[8] Alesci A., Aragona M., Cicero N., Lauriano E.R. (2022). Can nutraceuticals assist treatment and improve COVID-19 symptoms?. Nat. Prod. Res..

[9] Houghton P.J. (2001). Old yet new—Pharmaceuticals from plants. J. Chem. Educ..

[10] Salehi B., Zucca P., Sharifi-Rad M., Pezzani R., Rajabi S., Setzer W.N., Varoni E.M., Iriti M., Kobarfard F., Sharifi-Rad J. (2018). Phytotherapeutics in cancer invasion and metastasis. Phytother. Res..

[11] Musthaba S.M., Baboota S., Ahmed S., Ahuja A., Ali J. (2009). Status of novel drug delivery technology for phytotherapeutics. Expert Opin. Drug Deliv..

[12] Cravotto G., Boffa L., Genzini L., Garella D. (2010). Phytotherapeutics: An evaluation of the potential of 1000 plants. J. Clin. Pharm. Ther..

[13] Dave V., Yadav R.B., Ahuja R., Yadav S. (2017). Formulation design and optimization of novel fast dissolving tablet of chlorpheniramine maleate by using lyophilization techniques. Bull. Fac. Pharm. Cairo Univ..

[14] Cragg G.M., Newman D.J. (2013). Natural products: A continuing source of novel drug leads. Biochim. Biophys. Acta BBA Gen. Subj..

[15] Zaynab M., Fatima M., Abbas S., Sharif Y., Umair M., Zafar M.H., Bahadar K. (2018). Role of secondary metabolites in plant defense against pathogens. Microb. Pathog..

[16] Rajput V.D., Singh R.K., Verma K.K., Sharma L., Quiroz-Figueroa F.R., Meena M., Gour V.S., Minkina T., Sushkova S., Mandzhieva S. (2021). Recent developments in enzymatic antioxidant defence mechanism in plants with special reference to abiotic stress. Biology.

[17] Gorlenko C.L., Kiselev H.Y., Budanova E.V., Zamyatnin Jr A.A., Ikryannikova L.N. (2020). Plant secondary metabolites in the battle of drugs and drug-resistant bacteria: New heroes or worse clones of antibiotics?. Antibiotics.

[18] Asimuddin M., Shaik M.R., Adil S.F., Siddiqui M.R.H., Alwarthan A., Jamil K., Khan M. (2020). Azadirachta indica based biosynthesis of silver nanoparticles and evaluation of their antibacterial and cytotoxic effects. J. King Saud Univ. Sci..

[19] Guerriero G., Berni R., Muñoz-Sanchez J.A., Apone F., Abdel-Salam E.M., Qahtan A.A., Alatar A.A., Cantini C., Cai G., Hausman J.-F. (2018). Production of plant secondary metabolites: Examples, tips and suggestions for biotechnologists. Genes.

[20] Jain C., Khatana S., Vijayvergia R. (2019). Bioactivity of secondary metabolites of various plants: A review. Int. J. Pharm. Sci. Res.

[21] Jones W.P., Kinghorn A.D. (2006). Extraction of plant secondary metabolites. Natural Products Isolation.

[22] De Silva G.O., Abeysundara A.T., Aponso M.M.W. (2017). Extraction methods, qualitative and quantitative techniques for screening of phytochemicals from plants. Am. J. Essent. Oils Nat. Prod..

[23] Shaik M., Albalawi G., Khan S., Khan M., Adil S., Kuniyil M., Al-Warthan A., Siddiqui M., Alkhathlan H., Khan M. (2016). “Miswak” based green synthesis of silver nanoparticles: Evaluation and comparison of their microbicidal activities with the chemical synthesis. Molecules.

[24] Matrose N.A., Obikeze K., Belay Z.A., Caleb O.J. (2021). Impact of spatial variation and extraction solvents on bioactive compounds, secondary metabolites and antifungal efficacy of South African Impepho [*Helichrysum odoratissimum* (L.) Sweet]. Food Biosci..

[25] Dai J., Mumper R.J. (2010). Plant phenolics: Extraction, analysis and their antioxidant and anticancer properties. Molecules.

[26] Ahmed E., Arshad M., Khan M.Z., Amjad M.S., Sadaf H.M., Riaz I., Sabir S., Ahmad N. (2017). Secondary metabolites and their multidimensional prospective in plant life. J. Pharmacogn. Phytochem..

[27] Žlabur J.Š., Žutić I., Radman S., Pleša M., Brnčić M., Barba F.J., Rocchetti G., Lucini L., Lorenzo J.M., Domínguez R. (2020). Effect of different green extraction methods and solvents on bioactive components of chamomile (*Matricaria chamomilla* L.) flowers. Molecules.

[28] Adnan M., Oh K.K., Azad M.O.K., Shin M.H., Wang M.-H., Cho D.H. (2020). Kenaf (*Hibiscus cannabinus* L.) leaves and seed as a potential source of the bioactive compounds: Effects of various extraction solvents on biological properties. Life.

[29] Chiavaroli A., Sinan K.I., Zengin G., Mahomoodally M.F., Bibi Sadeer N., Etienne O.K., Cziáky Z., Jekő J., Glamočlija J., Soković M. (2020). Identification of chemical profiles and biological properties of Rhizophora racemosa G. Mey. extracts obtained by different methods and solvents. Antioxidants.

[30] Khan M., Khan M., Al-Hamoud K., Adil S.F., Shaik M.R., Alkhathlan H.Z. (2022). Comprehensive Phytochemical Analysis of Various Solvent Extracts of Artemisia judaica and Their Potential Anticancer and Antimicrobial Activities. Life.

[31] Hussain A.I., Rathore H.A., Sattar M.Z., Chatha S.A., Sarker S.D., Gilani A.H. (2014). *Citrullus colocynthis* (L.) Schrad (bitter apple fruit): A review of its phytochemistry, pharmacology, traditional uses and nutritional potential. J. Ethnopharmacol..

[32] Li Q.-Y., Munawar M., Saeed M., Shen J.-Q., Khan M.S., Noreen S., Alagawany M., Naveed M., Madni A., Li C.-X. (2021). *Citrullus colocynthis* (L.) Schrad (Bitter Apple Fruit): Promising traditional uses, pharmacological effects, aspects, and potential applications. Front. Pharmacol..

[33] Pravin B., Tushar D., Vijay P., Kishanchnad K. (2013). Review on *Citrullus colocynthis*. Int. J. Res. Pharm. Chem.

[34] Rahimi R., Amin G., Ardekani M.R.S. (2012). A review on *Citrullus colocynthis* Schrad.: From traditional Iranian medicine to modern phytotherapy. J. Altern. Complement. Med..

[35] Marzouk B., Marzouk Z., Haloui E., Fenina N., Bouraoui A., Aouni M. (2010). Screening of analgesic and anti-inflammatory activities of *Citrullus colocynthis* from southern Tunisia. J. Ethnopharmacol..

[36] Hameed B., Ali Q., Hafeez M., Malik A. (2020). Antibacterial and antifungal activity of fruit, seed and root extracts of *Citrullus colocynthis* plant. Biol. Clin. Sci. Res. J..

[37] Ostovar M., Akbari A., Anbardar M.H., Iraji A., Salmanpour M., Ghoran S.H., Heydari M., Shams M. (2020). Effects of *Citrullus colocynthis* L. in a rat model of diabetic neuropathy. J. Integr. Med..

[38] Cavazos P., Gonzalez D., Lanorio J., Ynalvez R. (2021). Secondary metabolites, antibacterial and antioxidant properties of the leaf extracts of *Acacia rigidula* benth. and Acacia berlandieri benth. SN Appl. Sci..

[39] Khan M., Khan S.T., Khan M., Mousa A.A., Mahmood A., Alkhathlan H.Z. (2019). Chemical diversity in leaf and stem essential oils of *Origanum vulgare* L. and their effects on microbicidal activities. AMB Express.

[40] Kurnia D., Ajiati D., Heliawati L., Sumiarsa D. (2021). Antioxidant properties and structure-antioxidant activity relationship of Allium species leaves. Molecules.

[41] Bourhia M., Bouothmany K., Bakrim H., Hadrach S., Salamatullah A.M., Alzahrani A., Khalil Alyahya H., Albadr N.A., Gmouh S., Laglaoui A. (2021). Chemical profiling, antioxidant, antiproliferative, and antibacterial potentials of chemically characterized extract of citrullus colocynthis L. seeds. Separations.

[42] Gurudeeban S., Ramanathan T., Satyavani K. (2011). Characterization of volatile compounds from bitter apple (*Citrullus colocynthis*) using GC-MS. Int. J. Chem. Anal. Sci..

[43] Singh S., Devi B. (2016). Estimation of phytoconstituents from *Citrullus colocynthis* (L.) schrad roots extract by GC-MS spectroscopy. Int. J. Sci. Res..

[44] El-Shazly A., Hafez S., Wink M. (2004). Comparative study of the essential oils and extracts of Achillea fragrantissima (Forssk.) Sch. Bip. and Achillea santolina L.(Asteraceae) from Egypt. Die Pharm. Int. J. Pharm. Sci..

[45] Alsohaili S.A., Al-fawwaz A.T. (2014). Composition and antimicrobial activity of Achillea fragrantissima essential oil using food model media. Eur. Sci. J..

[46] Alsohaili S. (2018). Seasonal variation in the chemical composition and antimicrobial activity of essential oil extracted from Achillea fragrantissima grown in Northern-Eastern Jordanian desert. J. Essent. Oil-Bear. Plants.

[47] Allenspach M., Valder C., Flamm D., Grisoni F., Steuer C. (2020). Verification of chromatographic profile of primary essential oil of Pinus sylvestris L. combined with chemometric analysis. Molecules.

[48] Allenspach M., Steuer C. (2021). α-Pinene: A never-ending story. Phytochemistry.

[49] Utegenova G.A., Pallister K.B., Kushnarenko S.V., Özek G., Özek T., Abidkulova K.T., Kirpotina L.N., Schepetkin I.A., Quinn M.T., Voyich J.M. (2018). Chemical composition and antibacterial activity of essential oils from *Ferula* L. species against methicillin-resistant *Staphylococcus aureus*. Molecules.

[50] Hou J., Zhang Y., Zhu Y., Zhou B., Ren C., Liang S., Guo Y. (2019). α-Pinene induces apoptotic cell death via caspase activation in human ovarian cancer cells. Med. Sci. Monit. Int. Med. J. Exp. Clin. Res..

[51] Marchese A., Orhan I.E., Daglia M., Barbieri R., Di Lorenzo A., Nabavi S.F., Gortzi O., Izadi M., Nabavi S.M. (2016). Antibacterial and antifungal activities of thymol: A brief review of the literature. Food Chem..

[52] Alagawany M., Farag M.R., Abdelnour S.A., Elnesr S.S. (2021). A review on the beneficial effect of thymol on health and production of fish. Rev. Aquac..

[53] Kachur K., Suntres Z. (2020). The antibacterial properties of phenolic isomers, carvacrol and thymol. Crit. Rev. Food Sci. Nutr..

[54] Kokkini S., Karousou R., Vokou D. (1994). Pattern of geographic variations of *Origanum vulgare* trichomes and essential oil content in Greece. Biochem. Syst. Ecol..

[55] Chorianopoulos N., Kalpoutzakis E., Aligiannis N., Mitaku S., Nychas G.-J., Haroutounian S.A. (2004). Essential oils of Satureja, Origanum, and Thymus species: Chemical composition and antibacterial activities against foodborne pathogens. J. Agric. Food. Chem..

[56] Sousa Silveira Z.d., Macêdo N.S., Sampaio dos Santos J.F., Sampaio de Freitas T., Rodrigues dos Santos Barbosa C., Júnior D.L.d.S., Muniz D.F., Castro de Oliveira L.C., Júnior J.P.S., Cunha F.A.B.d. (2020). Evaluation of the antibacterial activity and efflux pump reversal of thymol and carvacrol against *Staphylococcus aureus* and their toxicity in *Drosophila melanogaster*. Molecules.

[57] Qoorchi Moheb Seraj F., Heravi-Faz N., Soltani A., Ahmadi S.S., Talebpour A., Afshari A.R., Ferns G.A., Bahrami A. (2022). Thymol has anticancer effects in U-87 human malignant glioblastoma cells. Mol. Biol. Rep..

[58] Mittermeier V.K., Dunkel A., Hofmann T. (2018). Discovery of taste modulating octadecadien-12-ynoic acids in golden chanterelles (*Cantharellus cibarius*). Food Chem..

[59] Shoge M., Amusan T. (2020). Phytochemical, antidiarrhoeal activity, isolation and characterisation of 11-octadecenoic acid, methyl ester isolated from the seeds of *Acacia nilotica* Linn. J. Biotechnol. Immunol..

[60] Dhar P., Chan P., Cohen D.T., Khawam F., Gibbons S., Snyder-Leiby T., Dickstein E., Rai P.K., Watal G. (2014). Synthesis, antimicrobial evaluation, and structure–activity relationship of α-pinene derivatives. J. Agric. Food. Chem..

[61] Freitas P.R., de Araújo A.C.J., dos Santos Barbosa C.R., Muniz D.F., da Silva A.C.A., Rocha J.E., de Morais Oliveira-Tintino C.D., Ribeiro-Filho J., da Silva L.E., Confortin C. (2020). GC-MS-FID and potentiation of the antibiotic activity of the essential oil of *Baccharis reticulata* (ruiz & pav.) pers. and α-pinene. Ind. Crops. Prod..

[62] Zhu Z., Min T., Zhang X., Wen Y. (2019). Microencapsulation of Thymol in Poly (lactide-co-glycolide)(PLGA): Physical and Antibacterial Properties. Materials.

[63] Khoshbakht T., Karami A., Tahmasebi A., Maggi F. (2020). The variability of thymol and carvacrol contents reveals the level of antibacterial activity of the Essential Oils from different accessions of *Oliveria decumbens*. Antibiotics.

[64] Almehdar H., Abdallah H.M., Osman A.-M.M., Abdel-Sattar E.A. (2012). In vitro cytotoxic screening of selected Saudi medicinal plants. J. Nat. Med..

[65] Jo H., Cha B., Kim H., Brito S., Kwak B.M., Kim S.T., Bin B.-H., Lee M.-G. (2021). α-pinene enhances the anticancer activity of natural killer cells via ERK/AKT pathway. Int. J. Mol. Sci..

[66] Seresht H.R., Albadry B.J., Al-mosawi A.K.M., Gholami O., Cheshomi H. (2019). The cytotoxic effects of thymol as the major component of trachyspermum ammi on breast cancer (MCF-7) cells. Pharm. Chem. J..

[67] Alkhathlan H., Khan M., Abdullah M., AlMayouf A., Badjah-Hadj-Ahmed A.Y., AlOthman Z., Mousa A. (2015). Anticorrosive assay-guided isolation of active phytoconstituents from *Anthemis pseudocotula* extracts and a detailed study of their effects on the corrosion of mild steel in acidic media. RSC Adv..

[68] Al-Hwaiti M.S., Alsbou E.M., Abu Sheikha G., Bakchiche B., Pham T.H., Thomas R.H., Bardaweel S.K. (2021). Evaluation of the anticancer activity and fatty acids composition of “Handal”(*Citrullus colocynthis* L.) seed oil, a desert plant from south Jordan. Food Sci. Nutr..

[69] Joshi G., Kaur J., Sharma P., Kaur G., Bhandari Y., Kumar R., Singh S. (2019). P53-mediated anticancer activity of *Citrullus colocynthis* extracts. Nat. Prod. J..

[70] Khan M., Al-Saleem M.S., Alkhathlan H.Z. (2016). A detailed study on chemical characterization of essential oil components of two Plectranthus species grown in Saudi Arabia. J. Saudi Chem. Soc..

[71] Acree T., Arn H. (2004). Gas Chromatography-Olfactometry (GCO) of Natural Products. Flavornet and Human Odor Space, Sponsored by DATU Inc. http://www.flavornet.org.

[72] NIST Mass Spectrometry Data Center W.E.W., Linstrom P.J., Mallard W.G. (2020). Director “Retention Indices”. NIST Chemistry WebBook; NIST Standard Reference Database Number, 69.

[73] Adams R.P. (2007). Identification of Essential Oil Components by Gas Chromatography/Mass Spectrometry.

[74] Swapnaja K.J.M., Yennam S., Chavali M., Poornachandra Y., Kumar C.G., Muthusamy K., Jayaraman V.B., Arumugam P., Balasubramanian S., Sriram K.K. (2016). Design, synthesis and biological evaluation of diaziridinyl quinone isoxazole hybrids. Eur. J. Med. Chem..

[75] Hansen M.B., Nielsen S.E., Berg K. (1989). Re-examination and further development of a precise and rapid dye method for measuring cell growth/cell kill. J. Immunol. Methods.

