# Supplementary material for: Diversity of Citrullus colocynthis (L.) Schrad Seeds Extracts: Detailed Chemical Profiling and Evaluation of Their Medicinal Properties
Source: Plants (Basel). 2023 Jan 26;12(3):567. doi: 10.3390/plants12030567 (PMC9919198; doi:10.3390/plants12030567)
Supplement: Supplementary file 1 [file plants-12-00567-s001.zip › plants-2131752-supplementary.pdf]

plants

MDPI

Supplementary File

Diversity of *Citrullus colocynthis* (L.) Schrad Seeds Extracts: Detailed Chemical Profiling and Evaluation of Their Medicinal Properties

Merajuddin Khan *, Mujeeb Khan, Khaleel Al-hamoud, Syed Farooq Adil, Mohammed Rafi Shaik and Hamad Z. Alkhathlan

Department of Chemistry, College of Science, King Saud University, P.O. Box 2455, Riyadh 11451, Saudi Arabia

* Correspondence: mkhan3@ksu.edu.sa; Tel.: +966-11-4675910

Scheme S1. Gas Chromatography (GC) and Gas Chromatography−Mass Spectrometry (GC-MS) Analysis of *C. colocynthis* seeds Extracts

GC–MS was performed on an Agilent single-quadrupole mass spectrometer with an inert mass selective detector (MSD-5975C detector, Agilent Technologies, USA) coupled directly to an Agilent 7890A gas chromatograph which was equipped with a split–splitless injector, a quickswap assembly, an Agilent model 7693 autosampler and a HP-5MS fused silica capillary column (5% phenyl 95% dimethylpolysiloxane, 30 m × 0.25 mm i.d., film thickness 0.25 μm, Agilent Technologies, USA). The column was operated using an injector temperature of 250°C and the following oven temperature profile: an isothermal hold at 50°C for 4 min, followed by a ramp of 4°C/min to 220°C, an isothermal hold for 2 min, a second ramp to 280°C at 20°C/min and finally an isothermal hold for 15 min.

Approximately 0.2 μl of each sample dissolved in suitable solvents such as diethyl ether and methanol was injected using the split injection mode; the split flow ratio was 10:1. The helium carrier gas was flowed at 1 ml/min. The GC–TIC profiles and mass spectra were obtained using the ChemStation data analysis software, version E-02.00.493 (Agilent). All mass spectra were acquired in the EI mode (scan range of m/z 45–600 and ionization energy of 70 eV). The temperatures of the electronic-impact ion source and the MS quadrupole were 230°C and 150°C, respectively. The MSD transfer line was maintained at 280°C for the nalysis. The GC analysis was performed on an Agilent GC-7890A dual-channel gas chromatograph (Agilent Technologies, USA) equipped with FID using nonpolar (HP-5MS) columns under the same conditions as described above. The detector temperature was maintained at 300°C for the analyses. The relative composition of the oil components was calculated on the basis of the GC–FID peak areas measured using the HP-5 MS column without using correction factor. Results are reported in Table 1 according to their elution order on the HP-5MS column.

Scheme S2. Linear retention indices (LRIs)

A mixture of a continuous series of straight-chain hydrocarbons, C7-C30 (49451-U, Supelco, Sigma-Aldrich, USA) was injected into nonpolar (HP-5MS) columns under the same conditions previously described for the oil samples to obtain the linear retention indices (LRIs) (also referred to as linear temperature programmed retention indices (LTPRI)) of the oil constituents provided in Table 1. The LRIs were computed using van den Dool and Kratz's equation.

Scheme S3. Identification of volatile components

The identification of different phytocomponents of the extracts of *C. colocynthis* seeds was done by matching their mass spectra with the library entries of mass spectra databases (WILEY 9th edition, NIST-14 MS library version 2.2, and Adams and Flavor libraries) as well as by comparing their mass spectra and linear retention indices (LRIs) with published data [1-3]. Moreover, identification of some compounds such as thymol, δ3-carene, α-pinene, *n*-hexadecanoic acid, caryophyllene oxide, (*Z*)-9-Octadecenoic acid methyl ester, and 8,11-Octadecadienoic acid, methyl ester were further confirmed by using co-injection analysis/or by comparing their LRIs values with their respective pure standard compounds injected under the same GC conditions as described in scheme S1 for *C. colocynthis* seeds extracts.

References

1. Adams, R., Identification of essential oil components by gas chromatography/mass spectrometry. *Identification of essential oil components by gas chromatography/mass spectrometry.* **2007**, (Ed. 4).
2. Acree, T.; Arn, H., Flavornet. Cornell University, NYSAES. New York. 2012.
3. Linstrom, P.; Mallard, W., NIST Chemistry WebBook, NIST Standard Reference Database Number 69, June 2005. *National Institute of Standards and Technology, Gaithersburg MD* **2005,** 20899.
